# Supplementary material for: Patterns of contraceptive use through later reproductive years: A cohort study of Australian women with chronic disease
Source: PLoS One. 2023 May 3;18(5):e0268872. doi: 10.1371/journal.pone.0268872 (PMC10155986; doi:10.1371/journal.pone.0268872)
Supplement: S1 Table — (DOCX) [file pone.0268872.s001.docx]

**S1 Table. Comparison of analysed sample to entire cohort at baseline survey (1996).**

| **Characteristic** | **Category** | **Analysed sample**  **n=8,030**  **n (%)** | **Entire cohort**  **n=14,247**  **n (%)** |
| --- | --- | --- | --- |
| **Sociodemographics** |  |  |  |
| Country of birth | Australia | 7,140 (88.9) | 12,277 (86.2) |
|  | Other English-speakign background | 386 (4.8) | 727 (5.1) |
|  | Non-English-speaking background | 473 (5.9) | 1,179 (8.3) |
|  | *Missing* | *31 (0.4)* | *64 (0.4)* |
| Area of residence | Major cities | 4,137 (51.5) | 7,380 (51.8) |
|  | Inner regional | 2,441 (30.4) | 4,307 (30.2) |
|  | Outer regional/remote/very remote | 1,449 (18.0) | 2,555 (17.9) |
|  | *Missing* | *3 (0.0)* | *5 (0.0)* |
| Education | No formal qualifications | 124 (1.5) | 408 (2.9) |
|  | School certificate/higher school certificate | 5,365 (66.8) | 9,619 (67.5) |
|  | Trade/apprentice/certificate/diploma | 1,418 (17.7) | 2,563 (18.0) |
|  | University/higher degree | 1,084 (13.5) | 1,576 (11.1) |
|  | *Missing* | *39 (0.5)* | *81 (0.6)* |
| Relationship status | Partnered | 1,791 (22.3) | 3,193 (22.4) |
|  | Unpartnered | 6,210 (77.3) | 10,984 (77.1) |
|  | *Missing* | *29 (0.4)* | *70 (0.5)* |
| ^+^Income management | Impossible/difficult always | 1,304 (16.2) | 2,624 (18.4) |
|  | Difficult sometimes | 2,612 (32.5) | 4,706 (33.0) |
|  | Not too bad/easy | 4,095 (51.0) | 6,865 (48.2) |
|  | *Missing* | *19 (0.2)* | *52 (0.4)* |
| **Health factors** |  |  |  |
| Smoking | Non-smoker | 4,288 (53.4) | 7,123 (50.0) |
|  | Ex-smoker | 1,180 (14.7) | 2,085 (14.6) |
|  | Current smoker | 2,255 (28.1) | 4,421 (31.0) |
|  | *Missing* | *307 (3.8)* | *618 (4.3)* |
| Body mass index | Underweight | 633 (7.9) | 1,218 (8.6) |
|  | Healthy | 5,091 (63.4) | 8,469 (59.4) |
|  | Overweight | 1,080 (13.5) | 1,921 (13.5) |
|  | Obese | 445 (5.5) | 802 (5.6) |
|  | *Missing* | *781 (9.7)* | *1,837 (12.9)* |
| **Reproductive health** |  |  |  |
| History of pregnancy | No | 7,664 (95.4) | 13,467 (94.5) |
|  | Yes | 189 (2.4) | 419 (2.9) |
|  | *Missing* | *177 (2.2)* | *361 (2.5)* |
| History of termination | No | 7,438 (92.6) | 13,003 (91.3) |
|  | Yes | 493 (6.1) | 992 (7.0) |
|  | *Missing* | *99 (1.2)* | *252 (1.8)* |
| Parity | Zero | 7,460 (92.9) | 13,387 (94.0) |
|  | One | 439 (5.5) | 660 (4.6) |
|  | Two | 114 (1.4) | 172 (1.2) |
|  | Three or more | 17 (0.2) | 28 (0.2) |
|  | *Missing* | *0 (0.0)* | *0 (0.0)* |
| Menstrual symptoms | No | 6,022 (75.0) | 10,468 (73.5) |
|  | Yes | 1995 (24.8) | 3736 (26.2) |
|  | *Missing* | *13 (0.2)* | *43 (0.3)* |
| History of PCOS* | No | 8,029 (100.0) | 13,498 (94.7) |
|  | Yes | 1 (0.0) | 3 (0.0%) |
|  | *Missing* | *0 (0.0)* | *746 (5.2)* |
| History of endometriosis | No | 8,017 (99.8) | 13,484 (94.6) |
|  | Yes | 13 (0.2) | 17 (0.1) |
|  | *Missing* | *0 (0.0)* | *746 (5.2)* |

*PCOS = Polycystic ovary syndrome

^+^As Health care card status was unavailable at Survey 1, income management was included as a substitute.
