# Supplementary material for: Patterns of contraceptive use through later reproductive years: A cohort study of Australian women with chronic disease
Source: PLoS One. 2023 May 3;18(5):e0268872. doi: 10.1371/journal.pone.0268872 (PMC10155986; doi:10.1371/journal.pone.0268872)
Supplement: S2 Table — (DOCX) [file pone.0268872.s002.docx]

**S2 Table. Latent status transition probabilities (tau estimates) from Time 1 (2006) to Time 2 (2012), and from Time 2 (2012) to Time 3 (2018).**

| **Time 1 (2006) to Time 2 (2012)** | | | | | | |
| --- | --- | --- | --- | --- | --- | --- |
|  |  | Time 2 (2012, aged 34-39) | | | | |
|  |  | Short-acting & condom | Condom & natural | Sterilisation & other | LARC | No contraception |
| Time 1 (2006, aged 28-33) | Short-acting & condom | 0.44† | 0.20 | 0.13 | 0.11 | 0.11 |
|  | Condom & natural | 0.12 | 0.51† | 0.17 | 0.13 | 0.08 |
|  | Sterilisation & other | 0.05 | 0.01 | 0.86† | 0.03 | 0.05 |
|  | LARC | 0.15 | 0.14 | 0.17 | 0.48† | 0.05 |
|  | No contraception | 0.12 | 0.26 | 0.18 | 0.12 | 0.32† |
| **Time 2 (2012) to Time 3 (2018)** | | | | | | |
|  |  | Time 3 (2018, aged 40-45) | | | | |
|  |  | Short-acting & condom | Condom & natural | Sterilisation & other | LARC | No contraception |
| Time 2 (2012, aged 34-39) | Short-acting & condom | 0.41† | 0.14 | 0.17 | 0.15 | 0.13 |
|  | Condom & natural | 0.04 | 0.54† | 0.18 | 0.12 | 0.11 |
|  | Sterilisation & other | 0.01 | 0.01 | 0.90† | 0.02 | 0.05 |
|  | LARC | 0.04 | 0.05 | 0.15 | 0.71† | 0.06 |
|  | No contraception | 0.10 | 0.22 | 0.14 | 0.11 | 0.43† |
| † transition probability ≥ 0.30  LARC = Long-acting reversible contraception. | | | | | | |
