# Supplementary material for: Patterns of contraceptive use through later reproductive years: A cohort study of Australian women with chronic disease
Source: PLoS One. 2023 May 3;18(5):e0268872. doi: 10.1371/journal.pone.0268872 (PMC10155986; doi:10.1371/journal.pone.0268872)
Supplement: S3 Table — (DOCX) [file pone.0268872.s003.docx]

**S3 Table. Summary of LTA model diagnostics for the 1973-78 ALSWH cohort.**

| **Number of latent statuses** | **Number of iterations** | **G^2^** | **AIC** | **BIC** |
| --- | --- | --- | --- | --- |
| 3 | 181 | 17636.97 | 17706.97 | 17951.66 |
| 4 | 357 | 12021.54 | 12131.54 | 12516.04 |
| 5 | 126 | 6941.96 | 7099.96 | 7652.25 |
| 6 | 138 | 6390.84 | 6604.84 | 7352.87 |
| 7 | 1642 | 4456.31 | 4734.31 | 5706.05 |
| 8 | 1785 | 2607.70 | 2957.70 | 4181.12 |

* Shade indicates selected optimal model
