# Supplementary material for: Patterns of contraceptive use through later reproductive years: A cohort study of Australian women with chronic disease
Source: PLoS One. 2023 May 3;18(5):e0268872. doi: 10.1371/journal.pone.0268872 (PMC10155986; doi:10.1371/journal.pone.0268872)
Supplement: S4 Table — (DOCX) [file pone.0268872.s004.docx]

**S4 Table. Latent status membership probabilities (delta estimates) for the five-status model.**

| **Latent Status** | **Latent status description** | **Time 1**  **(2006)** | **Time 2**  **(2012)** | **Time 3**  **(2018)** |
| --- | --- | --- | --- | --- |
| Status 1 | Short-acting and condom | 0.44 | 0.26 | 0.15 |
| Status 2 | Condom and natural | 0.31 | 0.31 | 0.23 |
| Status 3 | Other and sterilisation | 0.08 | 0.03 | 0.04 |
| Status 4 | LARC | 0.05 | 0.14 | 0.21 |
| Status 5 | No contraception | 0.12 | 0.26 | 0.37 |

LARC = Long-acting reversible contraception.
