# Supplementary material for: Patterns of contraceptive use through later reproductive years: A cohort study of Australian women with chronic disease
Source: PLoS One. 2023 May 3;18(5):e0268872. doi: 10.1371/journal.pone.0268872 (PMC10155986; doi:10.1371/journal.pone.0268872)
Supplement: S9 Table — (DOCX) [file pone.0268872.s009.docx]

**S9 Table. Full multinomial mixed-effect models for factors associated with contraceptive use among Australian women (2006-2018), examining the impact of autoinflammatory disease.**

|  | **Condom and natural** | | | **Sterilisation and other** | | | **LARC** | | | **No contraception** | | |
| --- | --- | --- | --- | --- | --- | --- | --- | --- | --- | --- | --- | --- |
| **Characteristic** | **OR** | **95% CI** | ***P*** | **OR** | **95% CI** | ***P*** | **OR** | **95% CI** | ***P*** | **OR** | **95% CI** | ***P*** |
| Autoinflammatory disease |  |  |  |  |  |  |  |  |  |  |  |  |
| No | Ref. | - | - | Ref. | - | - | Ref. | - | - | Ref. | - | - |
| Yes | 1.20 | 1.00 - 1.44 | 0.04 | 1.61 | 1.08 - 2.39 | 0.02 | 1.18 | 0.91 - 1.53 | 0.21 | 1.32 | 1.04 - 1.66 | 0.02 |
| Age (years) | 1.08 | 1.04 - 1.11 | <0.001 | 1.39 | 1.28 - 1.51 | <0.001 | 1.08 | 1.03 - 1.13 | <0.001 | 1.14 | 1.09 - 1.19 | <0.001 |
| Country of birth |  |  |  |  |  |  |  |  |  |  |  |  |
| Australia | Ref. | - | - | Ref. | - | - | Ref. | - | - | Ref. | - | - |
| Other ESB | 1.37 | 1.12 - 1.69 | <0.001 | 0.82 | 0.46 - 1.45 | 0.48 | 1.28 | 0.91 - 1.80 | 0.16 | 0.99 | 0.72 - 1.36 | 0.95 |
| Non-ESB | 1.76 | 1.45 - 2.15 | <0.001 | 0.46 | 0.26 - 0.81 | 0.01 | 1.71 | 1.24 - 2.35 | 0.00 | 1.70 | 1.30 - 2.22 | <0.001 |
| Area of residence |  |  |  |  |  |  |  |  |  |  |  |  |
| Major cities | Ref. | - | - | Ref. | - | - | Ref. | - | - | Ref. | - | - |
| Inner regional | 0.91 | 0.81 - 1.02 | 0.10 | 2.29 | 1.76 - 2.98 | <0.001 | 1.06 | 0.89 - 1.27 | 0.50 | 1.05 | 0.90 - 1.23 | 0.54 |
| Outer regional/remote/very remote | 0.89 | 0.78 - 1.02 | 0.09 | 2.55 | 1.85 - 3.52 | <0.001 | 1.18 | 0.96 - 1.45 | 0.12 | 1.04 | 0.86 - 1.26 | 0.68 |
| Education |  |  |  |  |  |  |  |  |  |  |  |  |
| No formal qualifications | Ref. | - | - | Ref. | - | - | Ref. | - | - | Ref. | - | - |
| School certificate/higher school certificate | 0.86 | 0.47 - 1.57 | 0.62 | 0.43 | 0.13 - 1.42 | 0.17 | 1.53 | 0.55 - 4.27 | 0.42 | 0.71 | 0.34 - 1.48 | 0.36 |
| Trade/apprenticeship/certificate/diploma | 0.95 | 0.52 - 1.74 | 0.87 | 0.38 | 0.12 - 1.25 | 0.11 | 1.58 | 0.57 - 4.39 | 0.38 | 0.72 | 0.34 - 1.49 | 0.37 |
| University/higher degree | 1.09 | 0.60 - 2.00 | 0.78 | 0.22 | 0.07 - 0.74 | 0.01 | 1.66 | 0.60 - 4.63 | 0.33 | 0.57 | 0.28 - 1.20 | 0.14 |
| Relationship status |  |  |  |  |  |  |  |  |  |  |  |  |
| Partnered | Ref. | - | - | Ref. | - | - | Ref. | - | - | Ref. | - | - |
| Unpartnered | 0.69 | 0.61 - 0.79 | <0.001 | 0.12 | 0.08 - 0.17 | <0.001 | 0.98 | 0.79 - 1.21 | 0.87 | 0.91 | 0.76 - 1.09 | 0.31 |
| Health care card holder status |  |  |  |  |  |  |  |  |  |  |  |  |
| No | Ref. | - | - | Ref. | - | - | Ref. | - | - | Ref. | - | - |
| Yes | 1.11 | 0.95 - 1.30 | 0.18 | 1.09 | 0.78 - 1.53 | 0.60 | 1.15 | 0.91 - 1.46 | 0.25 | 1.16 | 0.94 - 1.43 | 0.17 |
| Smoking status |  |  |  |  |  |  |  |  |  |  |  |  |
| Non-smoker | Ref. | - | - | Ref. | - | - | Ref. | - | - | Ref. | - | - |
| Ex-smoker | 1.06 | 0.95 - 1.18 | 0.31 | 1.11 | 0.85 - 1.45 | 0.42 | 0.92 | 0.77 - 1.09 | 0.33 | 1.09 | 0.93 - 1.27 | 0.28 |
| Current smoker | 0.97 | 0.84 - 1.11 | 0.65 | 1.34 | 0.94 - 1.91 | 0.10 | 0.97 | 0.77 - 1.22 | 0.77 | 1.06 | 0.87 - 1.30 | 0.54 |
| Body Mass Index |  |  |  |  |  |  |  |  |  |  |  |  |
| Healthy weight | Ref. | - | - | Ref. | - | - | Ref. | - | - | Ref. | - | - |
| Underweight | 1.27 | 0.93 - 1.72 | 0.13 | 2.52 | 1.28 - 4.98 | 0.01 | 0.99 | 0.57 - 1.73 | 0.98 | 2.44 | 1.66 - 3.60 | <0.001 |
| Overweight | 0.94 | 0.84 - 1.04 | 0.23 | 1.07 | 0.84 - 1.36 | 0.60 | 1.01 | 0.86 - 1.20 | 0.86 | 1.12 | 0.96 - 1.30 | 0.17 |
| Obese | 0.88 | 0.78 - 1.00 | 0.05 | 1.00 | 0.75 - 1.32 | 0.98 | 1.29 | 1.07 - 1.56 | 0.01 | 1.22 | 1.03 - 1.44 | 0.02 |
| History of pregnancy |  |  |  |  |  |  |  |  |  |  |  |  |
| No | Ref. | - | - | Ref. | - | - | Ref. | - | - | Ref. | - | - |
| Yes | 1.41 | 1.22 - 1.62 | <0.001 | 7.57 | 5.40 - 10.60 | <0.001 | 2.56 | 2.02 - 3.23 | <0.001 | 0.74 | 0.61 - 0.90 | <0.001 |
| History of termination |  |  |  |  |  |  |  |  |  |  |  |  |
| No | Ref. | - | - | Ref. | - | - | Ref. | - | - | Ref. | - | - |
| Yes | 1.28 | 1.13 - 1.44 | <0.001 | 1.23 | 0.92 - 1.64 | 0.17 | 1.14 | 0.94 - 1.38 | 0.18 | 1.41 | 1.19 - 1.66 | <0.001 |
| Menstrual symptoms |  |  |  |  |  |  |  |  |  |  |  |  |
| No | Ref. | - | - | Ref. | - | - | Ref. | - | - | Ref. | - | - |
| Yes | 2.79 | 2.43 - 3.20 | <0.001 | 6.40 | 4.96 - 8.25 | <0.001 | 1.31 | 1.07 - 1.62 | 0.01 | 2.37 | 1.98 - 2.85 | <0.001 |
| History of PCOS |  |  |  |  |  |  |  |  |  |  |  |  |
| No | Ref. | - | - | Ref. | - | - | Ref. | - | - | Ref. | - | - |
| Yes | 1.02 | 0.77 - 1.35 | 0.91 | 0.70 | 0.37 - 1.34 | 0.28 | 0.86 | 0.56 - 1.32 | 0.50 | 1.72 | 1.24 - 2.38 | <0.001 |
| History of endometriosis |  |  |  |  |  |  |  |  |  |  |  |  |
| No | Ref. | - | - | Ref. | - | - | Ref. | - | - | Ref. | - | - |
| Yes | 0.74 | 0.57 - 0.94 | 0.02 | 0.54 | 0.29 - 0.98 | 0.04 | 1.29 | 0.90 - 1.84 | 0.17 | 1.02 | 0.75 - 1.40 | <0.001 |
| Time |  |  |  |  |  |  |  |  |  |  |  |  |
| Survey 4 | Ref. | - | - | Ref. | - | - | Ref. | - | - | Ref. | - | - |
| Survey 6 | 0.71 | 0.56 - 0.90 | <0.001 | 0.50 | 0.28 - 0.88 | 0.02 | 1.32 | 0.91 - 1.93 | 0.15 | 1.40 | 1.01 - 1.95 | 0.04 |
| Survey 8 | 0.67 | 0.45 - 1.00 | 0.05 | 0.42 | 0.15 - 1.14 | 0.09 | 2.14 | 1.13 - 4.06 | 0.02 | 1.66 | 0.95 - 2.92 | 0.08 |

Notes: ESB = English-speaking background; PCOS = Polycystic ovarian syndrome; Reference status = short-acting and condom; Parity was not included in the models due to overlap with pregnancy history.
