# Supplementary material for: Patterns of contraceptive use through later reproductive years: A cohort study of Australian women with chronic disease
Source: PLoS One. 2023 May 3;18(5):e0268872. doi: 10.1371/journal.pone.0268872 (PMC10155986; doi:10.1371/journal.pone.0268872)
Supplement: Attachment — Submitted filename: Response to Reviewers Document PONE-D-22-13508 (2023-04-03).doc [file pone.0268872.s010.doc]

| **Reviewer comments** | **Authors’ reply** |
| --- | --- |
| **Reviewer #1** |  |
| Overall, this is a well-written manuscript. | Thank you. |
| While this study is based on the Australian longitudinal study on women’s health, the current study is clearly retrospective. The title should not include “a prospective cohort study. | We have removed the term ‘prospective’ from the title. |
| I recommend clearly stating the study design in the methods section as well. From the description in the methodology section, this is a retrospective cohort study. | We have added the term ‘retrospective’ to line 98 to emphasise that the analysis was retrospective (i.e. the data was not collected with this specific analysis in mind). |
| For table 2, the presentation of the results should be changed such that the proportions are out of participants with available data. Missing data should not be part of the presented data. Instead, the authors should report on the proportion with available data for each variable. As an example for country of origin (without chronic disease), data is available for 3,990 participants of which 3,548 (88.9%) were born in Australia. | We have re-calculated the percentages to exclude missing data in the denominator. We have added a statement about the percentage of missing data to the notes of Table 2. It reads: *“missing data for each variable was less than 0.5%, except for history of pregnancy (0.52%), menstrual symptoms (0.68%) and body mass index (2.52%)*.” |
| It is also unclear whether there is any missing data for the chronic diseases in table 1. | We have added a statement about missing data for chronic diseases to the notes of Table 1. It reads: *“no missing data for chronic disease variables as these were derived from a range of data sources.”* |
| I recommend restructuring the discussion such that the strengths and limitations are at the end of the discussion. | As requested, the strengths and limitation section have been moved to the end of the discussion, above the conclusion. |
| The conclusion seems to suggest that there is a difference in contraception use between females with chronic diseases and those without. | The conclusion has been checked and clarified (see reponse below). Here we argue that while women with chronic disease take up contraception at similar rates to women without chronic disease, women with certain chronic diseases (i.e., autoinflammatory conditions) take up lower efficacy contraception at higher rates or did not to use contraception at all. |
| The conclusion should address the objectives. What are the patterns of contraceptive use in this cohort? How does chronic disease influence contraceptive use over time? The recommendations are not necessarily based on the results. | *The conclusion has been revised in line with the reviewer comments. It reads:*  *Across all women, the use of highly effective contraception such as LARC increased over time, although rates were still relatively low and contraceptive non-use also increased. This is problematic and highlights the general need for contraceptive care and counselling to reduce the risks of unintended pregnancy for all women, including those at later reproductive life. We also demonstrated that women with chronic disease take up contraception at similar rates to their same aged peers in the community. However, among women with chronic disease, women diagnosed with AICs were more likely to engage in low efficacy methods of contraception or did not use contraception. Our study therefore highlights the potential gaps in the provision of appropriate contraceptive access and care for women with chronic disease, particularly around the suitability of estrogen-containing contraceptives, including those diagnosed with AICs. The need for the development of national guidelines and a clear contraceptive strategy from adolescence through to menopause are required. Guidelines should encourage regular contraceptive review during routine care as well as training and education for medical professionals to increase support for, and agency among, women with chronic disease. This, in turn, will reduce the occurrence of high-risk unintended pregnancies and facilitate optimal outcomes for planned pregnancies.* |
| **Reviewer #2** |  |
| Congratulations to all authors! Great work! Background section is very well written/presented in the manuscript. It clearly highlights why this was a much needed research study/analysis. I also like the way the discussion section is framed.  Comments/questions that require authors' attention: | Thank you for your kind words. |
| Results section include comparison of groups on various factors and also specifies p-values (bracketed with many findings) to highlight the statistical significance of findings. However, the level of significance (aka. alpha level) at which these p-values were considered to be statistically significant is not clear. For example, was it alpha of 0.05 or 0.1 or 0.3? and why? | Apologies for the confusion, however there are no P-values in the results of the main manuscript. These are related to the probabilities of transitioning between latent statuses. We have now clarified this in the manuscript. |
| Q.1 Are all p-values mentioned under the heading of "Contraceptive use patterns over time", between line 268-275, considered and presented as "Statistically significant" by authors? If yes/no, please clarify (in either case). | As indicated above, these values relate to the probabilities of transitioning between latent statuses over time. This has been clarified by updating the manuscript to read ‘probability=’ rather than ‘P=’. |
| Q.2 Line 269-270 states, "women in Status 3 (“sterilisation and other”) in 2006 the most 270 likely to continue using the same contraception in 2012 (P=0.86)". Please clarify how the scientific community/readers of your manuscript should interpret "p=0.86" in terms of statistical significance of a finding stated in line 269-270, to highlight the strength of your evidence/data. | This confusion may have resulted from us not making it clear that ‘P’ indicates ‘Probability’ (not P-value). This sentence means that there is an 86% probability that women in Status 3 in 2006 would also be in Status 3 when measured again in 2012. The PROC LTA software used does not allow for an estimation of uncertainty around this transition probability, so we unfortunately cannot provide a confidence interval around this probability. However, given the relatively large sample sizes involved, we are comfortable presenting this result. |
| It seems like Table 2 is meant to show results of comparison between two groups of women - "with" and "without" chronic disease in this sample, but it doesn't include p-values or alpha level indicating at which 'level of significance' the found differences were meant to be considered statistically significant. IF available, I would suggest adding the same for all results/tables (where applicable). | We opted not to present P-values in this table, and generally avoided using them throughout the paper, based on the long-standing advice of the American Statistical Association and other prominent statisticians that over-reliance on P-values is highly problematic. Rather than relying heavily on P-values throughout the manuscript, we have instead opted to present confidence intervals wherever possible, which are considered a better measure of the degree of evidence for an association. |
| Also, consider indicating <0.01 p-values using numbers within the table, rather than using "-" and then adding a note to clarify the meaning of "-", since most of the readers tend to focus on highlighted/important numerical values and ignore '-'. | In Table 4, the values are not P-values, rather they are the item-response probabilities (as denoted in the column heading) that are standard in any paper that includes an LCA or LTA model. For example, for a participant in Status 1, they have a 0.15 probability of indicating ‘condom’ as a contraceptive. We opted to use dashes to achieve maximum clarity and avoid visual clutter where the item-response probabilities were very low and could effectively be ignored. We have clarified the meaning of the dashes in the note of Table 4. It reads: *“Dashed cells have item-response probabilities <0.01.”* |
| Thank you, PLOS ONE, for the opportunity to review such a great piece of work by brilliant researchers/authors! | Thank you very much. |
